# Supplementary material for: Synthesis and Evaluation of Peptide–Manganese Dioxide Nanocomposites as Adsorbents for the Removal of Strontium Ions
Source: Nanomaterials (Basel). 2023 Dec 23;14(1):52. doi: 10.3390/nano14010052 (PMC10780728; doi:10.3390/nano14010052)
Supplement: Supplementary file 1 [file nanomaterials-14-00052-s001.zip › nanomaterials-2776978-supplementary.pdf]

Supporting Information for

Synthesis and Evaluation of Peptide–Manganese Dioxide Nanocomposites as Adsorbents for the Removal of Strontium Ions

Xingjie Lu [1,2], Zhen Liu [1], Wentao Wang [2], Xin Wang [1], Hongchao Ma [1,*] and Meiwen Cao [1,*]

[1] State Key Laboratory of Heavy Oil Processing and Department of Biological and Energy Chemical Engineering, College of Chemical Engineering, China University of Petroleum (East China), 66 Changjiang West Road, Qingdao 266580, China

[2] Department of Radiochemistry, China Institute of Atomic Energy, Beijing 102413, China

[*] Correspondence: mahc@upc.edu.cn (H.M.); mwcao@upc.edu.cn (M.C.)

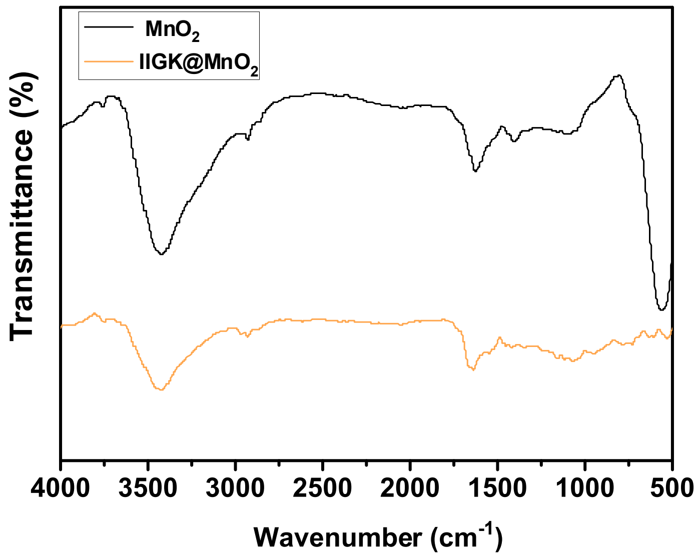

Figure S1. FTIR spectra of IIGK@MnO_2 and MnO_2.
